# Value of flow cytometry for MRD-based relapse prediction in B-cell precursor ALL in a multicenter setting

**DOI:** 10.1038/s41375-020-01100-5

**Published:** 2020-12-14

**Authors:** S. Modvig, H. Hallböök, H. O. Madsen, S. Siitonen, S. Rosthøj, A. Tierens, V. Juvonen, L. T. N. Osnes, H. Vålerhaugen, M. Hultdin, R. Matuzeviciene, M. Stoskus, M. Marincevic, A. Lilleorg, M. Ehinger, U. Norén-Nystrøm, N. Toft, M. Taskinen, O. G. Jónsson, K. Pruunsild, G. Vaitkeviciene, K. Vettenranta, B. Lund, J. Abrahamsson, A. Porwit, K. Schmiegelow, H. V. Marquart

**Affiliations:** 1 Department of Clinical Immunology, Copenhagen University Hospital Rigshospitalet, Copenhagen, Denmark; 2 Department of Medical Sciences, Uppsala University, Uppsala, Sweden; 3 Clinical Chemistry, University of Helsinki and Helsinki University Hospital, Helsinki, Finland; 4 Section of Biostatistics, University of Copenhagen, Copenhagen, Denmark; 5 Laboratory Medicine Program, University Health Network and University of Toronto, Toronto, ON Canada; 6 Department of Pathology, University Hospital of Oslo, Oslo, Norway; 7 Department of Clinical Chemistry and Laboratory Division, University of Turku and Turku University Hospital, Turku, Finland; 8 Department of Immunology, Oslo University Hospital, Oslo, Norway; 9 Department of Pathology, Laboratory of Molecular Pathology, Oslo University Hospital, Oslo, Norway; 10 Department of Medical Biosciences, Pathology, Umeå University, Umeå, Sweden; 11 Department of Physiology, Biochemistry, Microbiology and Laboratory Medicine, Institute of Biomedical Sciences, Faculty of Medicine, Vilnius University and Centre of Laboratory Medicine, Vilnius University Hospital Santaros Klinikos, Vilnius, Lithuania; 12 Hematology, Oncology and Transfusion Medicine Centre, Vilnius University Hospital Santaros Klinikos, Vilnius, Lithuania; 13 Department of Clinical Pathology, Uppsala University, Uppsala, Sweden; 14 Department of Clinical Immunology, North Estonia Medical Centre, Tallinn, Estonia; 15 Department of Clinical Genetics and Pathology, Skåne University Hospital and Department of Clinical Sciences, Oncology and Pathology, Lund University, Lund, Sweden; 16 Department of Clinical Sciences, Pediatrics, Umeå University, Umeå, Sweden; 17 Department of Hematology, Herlev University Hospital, Herlev, Denmark; 18 Division of Pediatric Hematology, Oncology and Stem Cell Transplantation, Helsinki University Hospital, Helsinki, Finland; 19 Children’s Hospital, Landspitali University Hospital, Reykjavik, Iceland; 20 Tallinn Children’s Hospital, Tallinn, Estonia; 21 Children’s Hospital, Affiliate of Vilnius University Hospital Santariskiu Klinikos, Vilnius, Lithuania; 22 University of Helsinki and Helsinki University Children´s Hospital, Helsinki, Finland; 23 Department of Pediatrics, St. Olavs University Hospital and Department of Clinical and Molecular Medicine, NTNU, Trondheim, Norway; 24 Institution of Clinical Sciences, Department of Pediatrics, Sahlgrenska University Hospital, Gothenburg, Sweden; 25 Division Oncology and Pathology, Department of Clinical Sciences, Faculty of Medicine, Lund University, Lund, Sweden; 26 Department of Pediatrics and Adolescent Medicine, Copenhagen University Hospital Rigshospitalet and The Institute of Clinical medicine, The Faculty of Medicine, University of Copenhagen, Copenhagen, Denmark

**Keywords:** Risk factors, Acute lymphocytic leukaemia, Translational research, Acute lymphocytic leukaemia

## Abstract

PCR of TCR/Ig gene rearrangements is considered the method of choice for minimal residual disease (MRD) quantification in BCP-ALL, but flow cytometry analysis of leukemia-associated immunophenotypes (FCM-MRD) is faster and biologically more informative. FCM-MRD performed in 18 laboratories across seven countries was used for risk stratification of 1487 patients with BCP-ALL enrolled in the NOPHO ALL2008 protocol. When no informative FCM-marker was available, risk stratification was based on real-time quantitative PCR. An informative FCM-marker was found in 96.2% and only two patients (0.14%) had non-informative FCM and non-informative PCR-markers. The overall 5-year event-free survival was 86.1% with a cumulative incidence of relapse (CIR_5y_) of 9.5%. FCM-MRD levels on days 15 (HzR 4.0, *p* < 0.0001), 29 (HzR 2.7, *p* < 0.0001), and 79 (HzR 3.5, *p* < 0.0001) associated with hazard of relapse adjusted for age, cytogenetics, and WBC. The early (day 15) response associated with CIR_5y_ adjusted for day 29 FCM-MRD, with higher levels in adults (median 2.4 × 10^−2^ versus 5.2 × 10^−3^, *p* < 0.0001). Undetectable FCM- and/or PCR-MRD on day 29 identified patients with a very good outcome (CIR_5y_ = 3.2%). For patients who did not undergo transplantation, day 79 FCM-MRD > 10^−4^ associated with a CIR_5y_ = 22.1%. In conclusion, FCM-MRD performed in a multicenter setting is a clinically useful method for MRD-based treatment stratification in BCP-ALL.

## Introduction

Minimal residual disease (MRD) is the single most important prognostic factor in acute lymphoblastic leukemia (ALL) [1–5], and guides the ALL post-induction treatment intensity. The current method of choice for MRD monitoring is real-time quantitative polymerase chain reaction of T-cell receptor (TCR)/immunoglobulin (Ig) gene rearrangements (PCR-MRD) [6]. However, not all patients with precursor B-ALL (BCP-ALL) have detectable gene rearrangements or sufficiently sensitive PCR markers, preventing proper risk stratification in 2–14% of patients [2, 7–9]. The possible loss of a marker during therapy requires the analysis of two PCR markers, which may further decrease the number of evaluable patients [2, 7, 8, 10]. Finally, PCR-MRD has a maximum sensitivity of 10^−5^ and most commonly a quantitative range (QR) of 10^−4^ due to the limit on DNA input [6]. Recently, high-throughput sequencing of TCR/Ig gene rearrangements (HTS-MRD) has emerged as a promising and sensitive method for MRD monitoring in BCP-ALL, but it is not yet fully validated for clinical use [11]. HTS-MRD has a relatively long turnaround time (4–5 days) and also requires a high DNA amount. Moreover, around 5% of the patients still remain without an identifiable MRD marker [11].

Flow cytometry is the alternative method used for MRD detection. It has a shorter turnaround time (1 day) and, in contrast to PCR, identifies the maturation stage and phenotypical heterogeneity of the leukemia. In addition, it provides information on the bone marrow cellular status. However, it is less sensitive than PCR, more difficult to standardize across centers, and requires a high level of expertise [6, 12]. Although comparative studies of FCM- versus PCR-MRD have been performed [8, 13–19], few study protocols have used FCM-MRD as the primary method for risk stratification in BCP-ALL. Ribera et al. reported on FCM-MRD-guided stratification to HSCT versus chemotherapy after early consolidation for 179 patients enrolled in five centers participating in the PETHEMA-ALL-AR-03 trial [20]. In the AALL0232 study by the Children’s Oncology Group FCM-MRD performed in two reference centers was used to risk stratify patients after first induction [21, 22]. Hitherto, no data are published on the clinical feasibility of FCM-MRD in an international, multicenter setting.

In this study, we show that FCM-MRD performed in 18 Nordic and Baltic centers is a valid approach for MRD detection at early and late stratifying time points providing prognostic value in relation to patient outcome in patients treated according to the Nordic Society of Pediatric Hematology and Oncology (NOPHO) ALL2008 protocol.

## Methods

### Subjects

In this study, we included 1298 children and 189 adults (<45 years of age) with BCP-ALL from Denmark, Estonia, Finland, Iceland, Lithuania, Norway, and Sweden, treated and monitored in the NOPHO ALL2008 protocol between July 1, 2008 and February 29, 2016 [23]. Patients with ambiguous lineage ALL [24], ALL predisposing syndromes, or pretreated with antileukemic agents, including corticosteroids, for >1 week prior to the diagnosis were excluded from the study [25]. In addition, patients with B-lymphoblastic lymphoma and Philadelphia chromosome positive BCP-ALL were excluded. The regional or national ethics committees approved the protocol, and informed consent was obtained according to the Declaration of Helsinki

### Protocol and MRD-based stratification

**Fig. 1:**
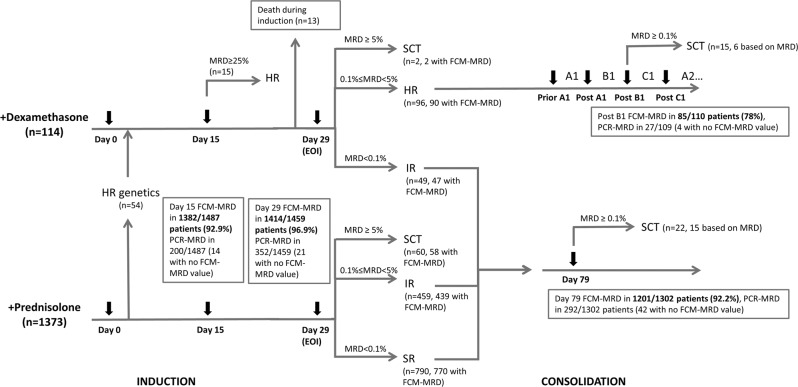
Stratification algorithm and MRD data for patients with BCP-ALL in the NOPHO ALL2008 protocol. Bone marrow sampling was performed on days 0, 15, and 29 (end of induction, EOI) of therapy, and on day 79 (for SR and IR patients) or prior to HR blocks until MRD was undetectable (for HR patients) (arrows). Patients with HR CG (*KMT2A*-r or hypodiploidy) entered the algorithm of patients in the +dexamethasone induction. Treatment stratification was performed at EOI to SR/IR/HR consolidation or HSCT. Patients with WBC <100 at diagnosis and no IR/HR CG (IR CG: dic(9;20), iAMP21 or t(1;19)) were stratified to SR by day 29 MRD <10^−3^ or to IR by MRD 10^−3^≤ to <5 × 10^−2^, while patients with WBC >100 and no HR CG were stratified to IR by MRD <10^−3^ or to HR by MRD 10^−3^≤ to <5 × 10^−2^. Stratification to SR/IR was not an option for patients with HR CG or to SR for those with IR CG. Patients with non-available day 29 MRD for non-HR cytogenetic groups entered the IR risk group at end of induction. Patients were stratified to HSCT if day 29 MRD was ≥5 × 10^−2^ or if day 79 (for SR/IR patients) or MRD after the second HR-block was ≥10^−3^. Further, patients with WBC ≥100 were stratified directly to HR-block therapy at day 15 by FCM-MRD ≥25%. Three patients had missing data for stratification at day 29. Eight patients were excluded from the day 29 analyses with the bone marrow sample date being >50 days from time of diagnosis. CG cytogenetic group, FCM-MRD flow cytometry-based minimal residual disease, HR high risk, IR intermediate risk, PCR-MRD polymerase-chain-reaction-based minimal residual disease, SCT hematopoietic stem cell transplantation, SR standard risk.

The NOPHO ALL2008 treatment and monitoring of BCP-ALL patients have been previously described [23]. In short, patients were stratified at diagnosis to induction therapy with either dexamethasone (10 mg/m^2^/day for 3 weeks) or prednisolone (60 mg/m^2^ for 4 weeks), weekly vincristine, two doses of doxorubicin (40 mg/m^2^ on days 1 and 22) with intrathecal methotrexate (or triple therapy for patients with central nervous system involvement). Bone marrow sampling was performed on days 0, 15, and 29 (end of induction, EOI) of therapy, and on day 79 (for standard risk (SR) and intermediate risk (IR) patients) or prior to high-risk (HR) blocks until MRD was undetectable (Fig. 1). Treatment stratification was performed on day 15 for +/− early HR-block therapy, on EOI (day 29) for SR/IR/HR consolidation or HSCT, and on day 79 (SR/IR) or after the second HR-block (HR) for +/− HSCT as described in Fig. 1.

### MRD analysis and retrospective revision of MRD data

MRD was measured by FCM-MRD using protocol-defined six-color panels for identification and monitoring of the leukemia-associated immunophenotype (LAIP) according to the NOPHO ALL2008 guidelines (Supplementary methods and Table S1), and by real-time quantitative PCR using clone-specific TCR/Ig primers according to the EuroMRD guidelines [26, 27]. All patients were evaluated for MRD marker informative status by both FCM and PCR at diagnosis. At MRD timepoints, the protocol specified that the first aspirate should be used for FCM- and PCR-MRD to avoid hemodilution and subsequent differences in assessment of blast concentration. The bone marrow material was split equally for FCM- and PCR-MRD. MRD-based treatment stratification was performed according to FCM-MRD, when an informative LAIP was identified. If not, patients were stratified by PCR-MRD, when specific and sufficiently sensitive (QR <10^−3^) markers were available. As a standard, two PCR-markers were required, but when not identified, one sensitive marker could be accepted. The FCM-MRD analysis was performed in 18 centers and PCR-MRD in 7 centers. Three centers performed FCM- and PCR-MRD in parallel at all timepoints.

FCM- and PCR-MRD results were defined as discrepant when it would result in a different risk stratification at 10^−3^ level on day 29 or detectable/undetectable. The latter was defined as undetectable MRD by one method but detectable ≥10^−4^ by the other, with a >2.5-fold higher value than the detection limit of the method with undetectable MRD, to account for the variation of the detection limits of the respective methods. For patients with discrepant day 29 FCM- and PCR-MRD results, FCM-files and PCR data were reviewed by experienced members of the Nordic flow cytometry group and the national PCR coordinators to explain the discrepancy.

### Statistical analysis

The main clinical outcomes of this study were 5-year event-free survival (EFS_5y_) and 5-year cumulative incidence of relapse (CIR_5y_). Survival analysis with time since diagnosis as the underlying timescale and delayed entry at time of MRD-measurement was used for analysis of the association between MRD and outcome. For analysis of outcome after HSCT, time since HSCT comprised the underlying timescale. For the analysis of day 15/29 MRD and prognosis, patients stratified directly to HSCT based on day 29 MRD were excluded, as this significantly changed their prognosis. Patients stratified to HSCT on day 79/after the second HR-block were censored at time of HSCT. Death and secondary malignancy were treated as competing events in the analyses of relapse, and for the day 15 analyses, induction failure was additionally considered a competing event. The Kaplan–Meier method was used to determine EFS, and the Aalen–Johansen estimator to determine CIR. Survival curves were compared using the log-rank test, and CIR_5y_ compared using the Wald test. Cause-specific Cox regression with delayed entry at time of MRD measurement was used to determine the association between MRD and the hazard of relapse adjusting for age group (<10 years, 10–17 years, ≥18 years), WBC (</≥100 × 10^9^/L), cytogenetic risk group (cytogenetic standard risk (SR CG): high hyperdiploidy (51–67 chromosomes) or t(12;21)(p13;q22)). Cytogenetic intermediate risk (IR CG): dic(9;20)(p11–13;q11) or t(1;19)(q23;p13), or intrachromosomal amplification of chromosome 21. Cytogenetic high risk (HR CG): *KMT2A* rearrangement (*KMT2A*-r) or hypodiploidy (23–39 chromosomes). B-other: patients with no stratifying genetic aberration, normal genetic findings, or with a not informative cytogenetic result), and in analyses of days 15 and 79 MRD also adjusting for day 29 MRD (</≥10^−3^). The proportional hazards assumption and linearity of quantitative variables were evaluated using tests based on sums of cumulative martingale residuals [28]. Spearman’s correlation coefficient was used to explore the association between FCM- and PCR-MRD values. Fisher’s exact test was used for comparison of categorical variables between groups. Cohen’s kappa for dichotomized MRD values, and Bland–Altman plots with limits of agreement for quantitative MRD values were used to assess agreement. CI represents 95% confidence intervals. *P* values < 0.05 were considered significant. Statistical analyses were performed in SAS version 9.4 and R version 3.6.0.

## Results

### Feasibility of FCM-MRD

Information on FCM or PCR marker informative status at diagnosis was registered in 1455 patients (Tables 1 and S2). Of these, an informative LAIP for MRD monitoring was identified in 1399 patients (96.2%). Of the remaining, 43 had an informative PCR marker. Only two patients (0.14% of total) had a non-informative LAIP and also lacked a sensitive antigen receptor gene rearrangement, while 11 lacked an informative marker due to other technical reasons or insufficient sample material (Table S2). Among the 31 patients with a non-informative LAIP, t(1;19) (*n* = 4, *p* = 0.01), dic(9;20) (*n* = 3, *p* = 0.02), and hypodiploidy (*n* = 3, *p* = 0.01) were overrepresented, while high hyperdiploidy was underrepresented (*n* = 2, *p* = 0.002) compared to remaining cases.

**Table 1:** Patient characteristics.

Clinical characteristics	
Age	4 (2–11, 1–45) years
Gender	781/706 (52.5/47.5%) male/female
White blood cell count (WBC) at diagnosis	9 (4.2–28.6, 0.4–1161) x10^9^/L
t(12;21)(p13;q22)	345 (23.2%)
High hyperdiploidy (51–67 chromosomes)	470 (31.6%)
t(1;19)(q23;p13)	47 (3.2%)
*KMT2A* rearrangement	52 (3.5%)
iAMP21	28 (1.9%)
dic(9;20)(p11–13;q11)	29 (2%)
Hypodiploidy (23–39 chromosomes)	23 (1.5%)
B-other (no stratifying genetic aberration or not informative^a^)	493 (33.2%)
CNS status at diagnosis	1326/114/42/5 (89/8/3/0%) CNS1/CNS2/CNS3/n.a.
Follow up time for patients in remission	51 (31–75, 0.6–103) months

For continuous variables, median (IQR, range) is given, for categorical variables, absolute number (percentage) is given. CNS1: no malignant blasts detected in the cerebrospinal fluid (CSF) by cytospin. CNS2: malignant blasts detected in the CSF by cytospin and CSF leukocyte count <5. CNS3: malignant blasts detected in the CSF by cytospin and CSF leukocyte count ≥5.

^a^For 40/493 patients classified as B-other, ≥1 of the cytogenetic tests were missing. There were no positive findings in the tests that were performed for this group and none lacked all analyses. Reperforming analyses excluding these patients from the B-other group did not change the conclusions of the study.

### EOI MRD determined by FCM and clinical outcome

The overall EFS_5y_ was 86.1% (CI 84.1–88.1%) with a CIR_5Y_ of 9.5% (CI 7.8–11.3%) in this cohort of 1487 patients (1298 children and 189 adults).

**Fig. 2:**
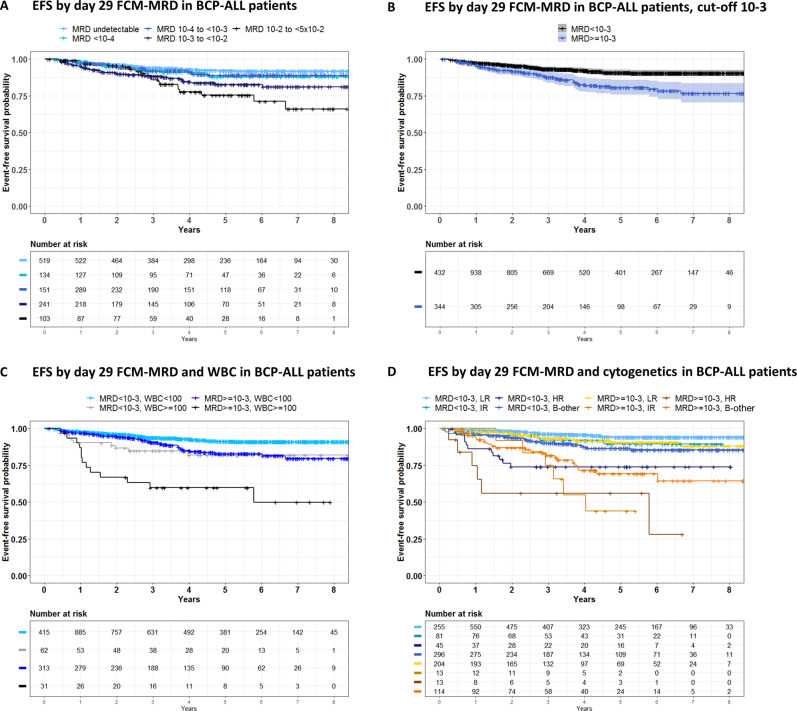
Day 29 FCM-MRD level, WBC, cytogenetics, and EFS. **A** EFS by day 29 FCM-MRD level. Patients are grouped by day 29 FCM-MRD in log10 intervals. **B** EFS and day 29 FCM-MRD level, cutoff level 10^−3^. **C** EFS by WBC (cutoff level 100 × 10^9^/L) and day 29 FCM-MRD (cutoff level 10^−3^). **D** EFS by cytogenetic group and day 29 FCM-MRD (cutoff level 10^−3^). SR CG comprise high hyperdiploidy and t(12;21) while IR CG comprise dic(9;20), iAMP21, and t(1;19). The HR CG comprises *KMT2A*-r and hypodiploidy. B-other comprises patients with no stratifying genetic aberration, normal genetic findings, or with a not informative cytogenetic result. CG cytogenetic group, CIR cumulative incidence of relapse, EFS event-free survival, HR high risk, IR intermediate risk, MRD minimal residual disease, SR standard risk.

The day 29 FCM-MRD was associated with EFS and CIR (Fig. 2A, B). In a multivariable analysis including the risk factors of the protocol, FCM-MRD >10^−3^ at EOI, WBC >100, older age groups, and IR/HR/B-other CG were all predictors of relapse (Table 2). Figure 2C illustrates the combined effects on EFS of WBC and FCM-MRD.

**Table 2:** Risk factors for relapse in BCP-ALL.

Variable	HzR (95% CI)	*p* value
EOI FCM-MRD > 10^−3^	2.7 (1.8–4.1)	<0.0001
WBC ≥ 100	2.6 (1.5–4.5)	0.0006
IR CG	2.9 (1.4–6.4)	0.006
HR CG	3.9 (1.7–9.3)	0.002
B-other	2.8 (1.7–4.8)	0.0001
Children 10–17 years	1.9 (1.1–3.3)	0.02
Adults 18–45 years	2.4 (1.4–4.1)	0.002

Multivariable cause-specific Cox regression analysis of time to relapse. Death and secondary malignancy were considered competing risks and censored at the time point of event. Reference groups: FCM-MRD < 10^−3^, WBC <100, SR CG and children <10 years. *N* = 1314. The SR CG comprises patients with high hyperdiploidy or t(12;21). The IR CG comprises patients with t(1;19), dic(9;20), or iAMP21. The HR CG comprises patients with *KMT2A*-r or hypodiploidy. B-other comprises patients with no stratifying genetic aberration, normal genetic findings, or with a not informative cytogenetic result.

*CG* cytogenetic group, *EOI* end of induction, *FCM-MRD* flow cytometry-based minimal residual disease, *HR* high risk, *IR* intermediate risk, *SR* standard risk, *WBC* white blood cell count.

In patients stratified to SR consolidation by FCM-MRD <10^−3^ (*n* = 767), we found no difference in EFS_5y_ for patients with FCM-MRD ≥10^−4^ (EFS_5y_ 92.1, CI 88.1–96.3, *n* = 527) and <10^−4^ (EFS_5y_ 91.1, CI 88.3–94.0, *n* = 240, Fig. 2A). For those stratified to IR consolidation by FCM-MRD <10^−3^ (initial WBC >100, *n* = 47), patients with FCM-MRD ≥10^−4^ had an EFS_5y_ of 77.8% (CI 57.1–100%, *n* = 18) versus EFS_5y_ 92.0 (CI 81.7–100%, n = 29) for those with FCM-MRD <10^−4^.

Patients with FCM-MRD of 10^−3^ to <5 × 10^−2^ on day 29 and B-other had a poor outcome (EFS_5y_ 69.3%, CI 59–81%, CIR_5Y_ 28.8%, CI 18–40%, Fig. 2D). This group consisted of 114 patients, of which 41 (36%) were adults. The B-other adult ALL-IR group contained 58 of the 84 adult ALL-IR patients and included all the 13 relapses among the adult ALL-IR patients (*p* = 0.0001, Wald test for comparison of CIR_5y_ for B-other versus non-B-other adult ALL-IR). Among the 17 adult ALL-IR patients with MRD <10^−3^, three experienced relapse.

### Undetectable MRD by FCM and/or PCR defines very good responders at EOI

Out of 331 patients with MRD results by both methods, 123 had undetectable day 29 MRD by FCM- and/or PCR with an overall CIR_5Y_ of 3.2% (CI 1.9–6.85). Among the 62 with undetectable day 29 MRD by both methods, two experienced relapse (CIR_5Y_ 4.9%, CI 0–11.8%, FCM-MRD sensitivity 6 × 10^−5^/8 × 10^−5^, PCR-MRD sensitivity 1 × 10^−5^/5 × 10^−4^ for the two). Both also had undetectable FCM- and PCR-MRD on day 79. Among 48 patients with undetectable FCM-MRD, but detectable PCR-MRD, there was one relapse (CIR_5Y_ 2.2%, CI 0–6.4%, FCM-MRD sensitivity 2 × 10^−5^), and none among the 13 with undetectable PCR-MRD and detectable FCM-MRD. There was no association between undetectable MRD with subsequent relapse and specific cytogenetic aberrations.

### Early response predicts clinical outcome

**Fig. 3:**
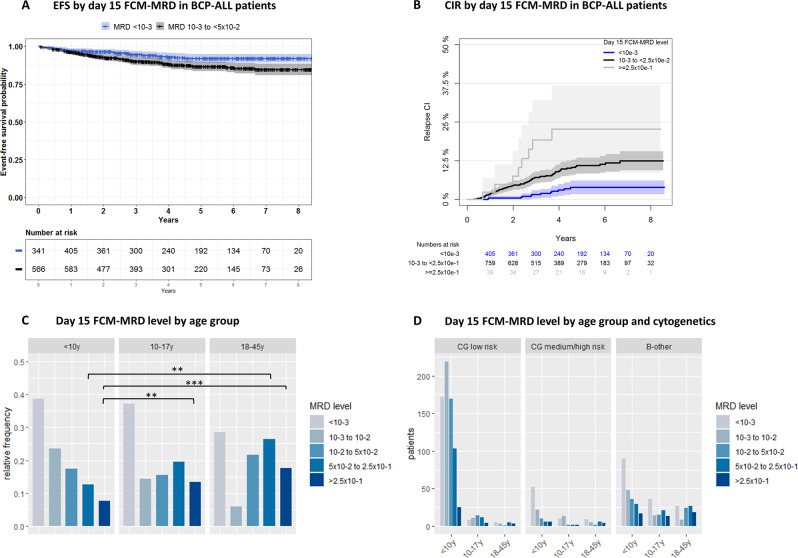
Day 15 FCM-MRD association with outcome and age. **A** EFS and day 15 FCM-MRD level, grouped by 10^−3^. **B** CIR and day 15 FCM-MRD level, grouped by 10^−3^ and 2.5 × 10^−1^, the latter being the cutoff level for an early stratification to HR risk therapy. **C** Day 15 FCM-MRD association with age. **p < 0.01, ***p < 0.001, Fischer’s exact test. **D** Day 15 FCM-MRD association with age by the cytogenetic risk group, showing that CG low risk in children <10 years as well as patients defined as B-other contribute the most to the age-related differences seen in **C**. CG low risk includes patients with high hyperdiploidy or t(12;21), while CG medium/high risk includes patients with dic(9;20), t(1;19), iAMP21, *KMT2A*-r, and hypodiploidy. B-other includes patients with no stratifying genetic aberration, normal genetic findings, or with a not informative cytogenetic result. CG cytogenetic group, CIR cumulative incidence of relapse, EFS event-free survival, MRD minimal residual disease.

The day 15 FCM-MRD level was associated with the outcome with clear differences in EFS and CIR using a cutoff level of 10^−3^. Patients with FCM-MRD <10^−3^ had an EFS_5y_ of 92.0% (CI 89.2–95.0%) and a CIR_5y_ of 3.9% (CI 1.7–6.1%, *n* = 432) whereas those with FCM-MRD ≥10^−3^ to <5 × 10^−2^ had an EFS_5y_ of 86.5% (CI 83.5–89.7%, *n* = 636, *p* = 0.0058) and a CIR_5y_ of 9.9% (CI 7.1–12.7%, *p* = 0.001, Fig. 3A, B). When adjusting for WBC, age, and CG, the day 15 FCM-MRD ≥10^−3^ to <5 × 10^−2^ had a hazard ratio (HzR) of 4.0 (CI 2.1–7.7) for relapse compared with FCM-MRD <10^−3^ (*p* < 0.0001). When including the day 29 FCM-MRD in a multivariable analysis, the day 15 response remained associated with the hazard of relapse (HzR 3.5 (CI 1.8–6.9) for FCM-MRD ≥10^−3^ to <5 × 10^−2^ on day 15 versus FCM-MRD <10^−3^, *p* = 0.0003).

The early response was associated with age (FCM-MRD median 2.4 × 10^−2^ in adults versus 5.2 × 10^−3^ in children, *p* < 0.0001) with adults having a significantly higher proportion of patients with MRD ≥5 × 10^−2^ than children <10 years on day 15 (Fig. 3C). This difference was related to cytogenetic subtypes with the SR CG subtypes being more frequent among the children <10 years and displaying low day 15 FCM-MRD, while the B-other group was most frequent among the older children and adults, showing clear differences in MRD levels among age groups (Fig. 3D).

### Persistent residual disease by FCM-MRD in non-transplanted SR/IR patients is associated with poor outcome

**Fig. 4:**
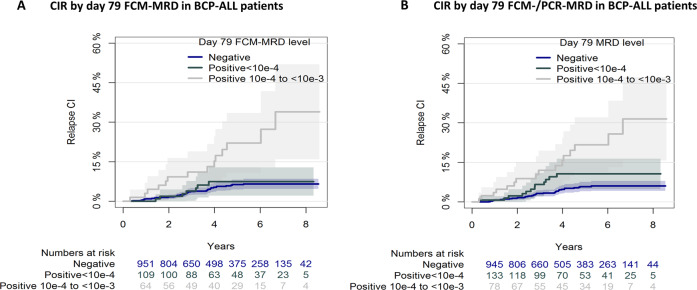
Day 79 MRD and outcome in patients not stratified for HSCT. **A** CIR by day 79 FCM-MRD in patients not stratified for HSCT (MRD <10^−3^) showing that patients with MRD levels of 10^−4^ to <10^−3^ on day 79 had a significantly higher CIR. **B** CIR by day 79 MRD in patients not stratified for HSCT. The higher value of FCM and PCR-MRD was selected, if results from both methods were available. Patients with an MRD of 10^−4^ to <10^−3^ on day 79 had a significantly higher CIR (21.7%, CI 11.3–32.1%, *n* = 86) compared to those with detectable MRD <10^−4^ (10.7%, CI 4.9–16.4%, *n* = 137) or undetectable MRD (5.8%, CI 4.0–7.6%, *n* = 987, *p* = 0.0047 for MRD of 10^−4^ to <10^−3^ versus <10^−4^/undetectable). CIR cumulative incidence of relapse, EFS event-free survival, FCM-MRD flow cytometry-based minimal residual disease, PCR-MRD polymerase-chain-reaction-based minimal residual disease.

In the ALL2008 protocol, patients were stratified to HSCT by MRD ≥10^−3^ on day 79 or after the second HR-block. We therefore examined the clinical outcome of patients with MRD <10^−3^ at these two timepoints. Patients with day 79 FCM-MRD of 10^−4^ to <10^−3^ had a significantly higher CIR_5y_ (22.1%, CI 10.8–33.5%, *n* = 68) compared with patients with day 79 FCM-MRD <10^−4^ (7.5%, CI 2.1–12.8%, *n* = 110) or undetectable (6.3%, CI 4.5–8.2%, *n* = 999, Fig. 4A, *p* = 0.0087 for FCM-MRD 10^−4^ to <10^−3^ versus <10^−4^/undetectable).

These findings were confirmed by day 79 PCR-MRD values. For patients with an undetectable PCR-MRD (*n* = 214), the CIR_5y_ (7.1%, CI 3.1–11.2%) was comparable to that of undetectable day 79 FCM-MRD. The number of patients with detectable MRD by PCR was too small for analyses (*n* = 35 with PCR-MRD <10^−4^, *n* = 29 with PCR-MRD of 10^−4^ to <10^−3^), and we thus combined FCM- and PCR-MRD results, selecting the higher value of the two. This confirmed that patients with MRD of 10^−4^ to <10^−3^ on day 79 had a significantly higher CIR (Fig. 4B).

After adjusting for WBC, age, and cytogenetics, the day 79 FCM-MRD remained associated with a higher hazard of relapse (HzR 3.5, CI 1.9–6.4, *p* < 0.0001) for day 79 FCM-MRD of 10^−4^ to <10^−3^ compared with undetectable FCM-MRD (*n* = 1171). Further adjusting for the day 29 FCM-MRD, the day 79 FCM-MRD remained associated with relapse (HzR 2.8, CI 1.5–5.3, *p* = 0.001 for FCM-MRD of 10^−4^ to <10^−3^ versus <10^−4^/undetectable).

After the second HR-block, no difference in outcome was demonstrated between patients with undetectable FCM-MRD (CIR_5Y_ 17.3%, CI 6.7–27.6%, *n* = 57) and patients with detectable FCM-MRD <10^−4^ (CIR_5Y_ 17.4% (CI 0–35.4%, *n* = 18). Only five patients had FCM-MRD of 10^−4^ to <10^−3^, two of whom experienced relapse. PCR-MRD results after the second HR block were available for 22 patients who did not undergo HSCT. Of these, 16 had undetectable MRD (0 relapses). Of five with PCR-MRD <10^−4^, three experienced relapse.

MRD >10^−3^ on day 79 was detected in 33 patients either by FCM- or PCR-MRD of which 15 underwent HSCT. Three of the non-transplanted patients and two of the transplanted patients experienced relapse. After the second HR-block, seven patients had FCM-MRD >10^−3^. Of these, one did not undergo HSCT and experienced relapse, and six underwent HSCT, of which four experienced relapse.

### MRD and outcome after HSCT

A total of 89 patients underwent HSCT in CR1, 74 of whom were transplanted based on a stratifying MRD value (48 by FCM only, 25 by concordant FCM and PCR, one by PCR only). The 89 had an overall EFS_5y_ from time of transplantation of 73.3% (CI 63.1–85.3%) with a CIR_5Y_ of 19.5% (CI 9.3–29.8%).

Only 9/89 HSCT patients were stratified early (day 15) to HR-block therapy based on a combination of HR cytogenetics/WBC >100 and day 15 MRD >2.5 × 10^−1^. These patients had a CIR_5Y_ after HSCT of 51.9% (CI 14.2–89.5%). In comparison, patients not stratified to early HR therapy had a CIR_5Y_ after HSCT of 15.9% (5.6–26.2%).

### FCM- and PCR-MRD results are highly comparable

**Fig. 5:**
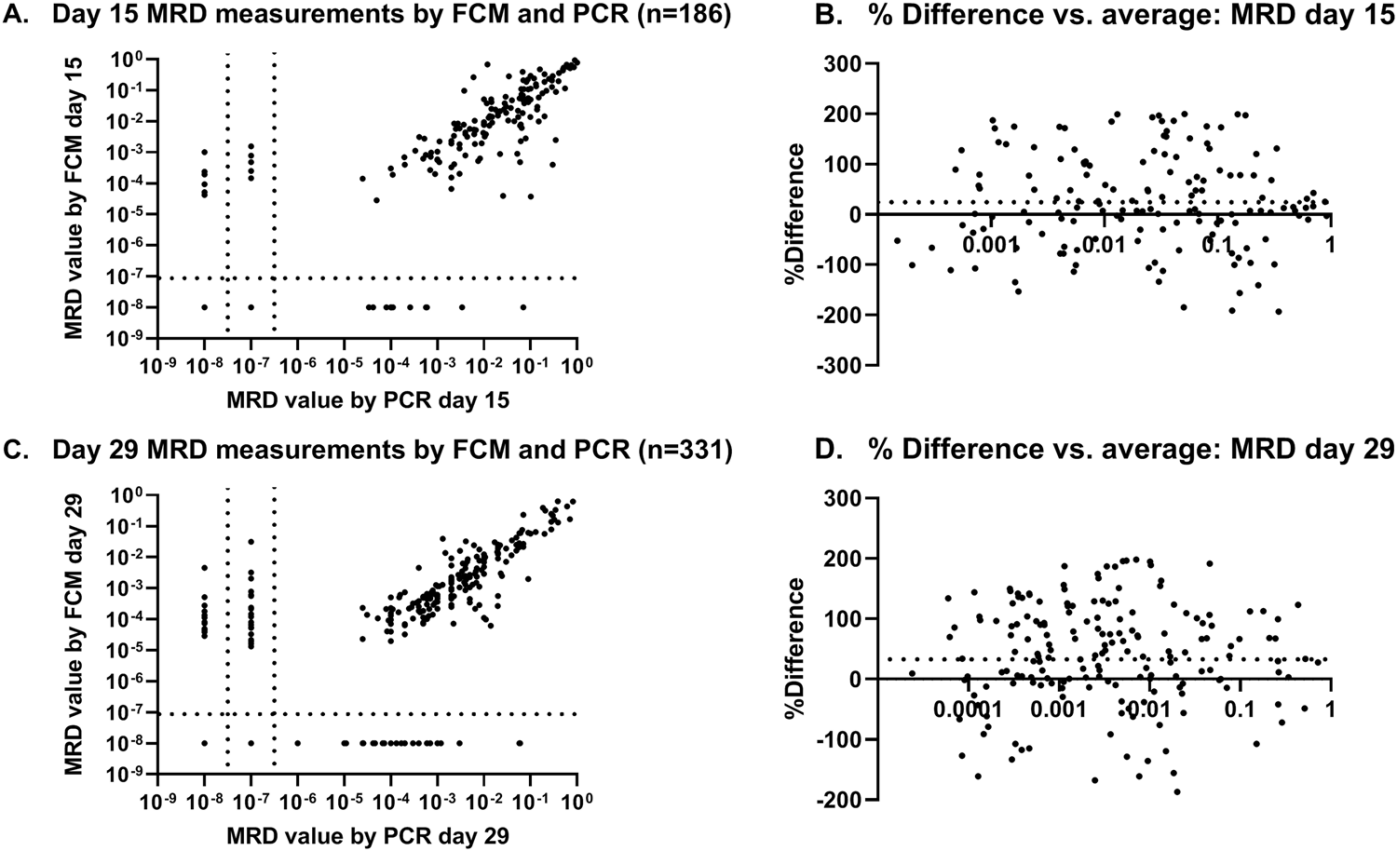
MRD measurements by FCM and PCR days 15 and 29. **A**, **B** Association between FCM- and PCR-MRD on day 15 (*n* = 186). Of the 186 patients, 153 had quantifiable MRD by both methods, which correlated strongly (*r* = 0.77, *p* < 0.0001). PCR-MRD was higher than FCM-MRD (mean 26% higher, limits of agreement −160–212%, Bland–Altman (**B**)). **C**, **D** Association between FCM- and PCR-MRD on day 29 (*n* = 331). MRD was detectable by both methods in 183 patients, showing a strong correlation (*r* = 0.83, *p* < 0.0001). PCR-MRD was higher than FCM-MRD (mean 42% higher, limits of agreement −123–207%, Bland–Altman (**D**)). FCM flow cytometry, LOD limit of detection, MRD minimal residual disease, PCR polymerase chain reaction, QR quantitative range.

PCR-MRD and FCM-MRD were performed in parallel in 352 samples taken at EOI. There was a strong correlation between FCM and PCR-MRD levels on days 15 (*r* = 0.77, *p* < 0.0001, *n* = 153) and 29 (*r* = 0.83, *p* < 0.0001, *n* = 183, Fig. 5). Furthermore, there was a good agreement between the methods around the day 29 stratification cutoff level of 10^−3^ (Cohen’s kappa 0.77, CI 0.69–0.84, *n* = 331). At later timepoints there were too few patients with quantifiable MRD by both methods for reliable comparison (day 79 *n* = 21, after the second HR-block *n* = 2).

On day 29, the percentage of patients with detected MRD by FCM of the patients with MRD detected by PCR (FCM_det_/PCR_det_) was 81.2%, whereas it was 94.1% for the reverse ratio (PCR_det_/FCM_det_), and the overall percentage of discordant cases was 18.1% (Fig. S1). FCM- and PCR-MRD median sensitivity/QR for stratifying time points is shown in Table S3. In total, FCM did not detect MRD in 559 patients on day 29. Median sensitivity for the patients without detectable FCM-MRD and with detectable FCM-MRD was comparable. (4.8 × 10^−5^ (IQR 3.1–8.8 × 10^−5^) versus 4.6 × 10^−^^5^ (IQR 3.0–7.4 × 10^−5^), *p* = 0.09, *n* = 1414). The FCM-MRD sensitivity increased by calendar year, indicating that more cells were acquired over the years of the protocol.

**Fig. 6:**
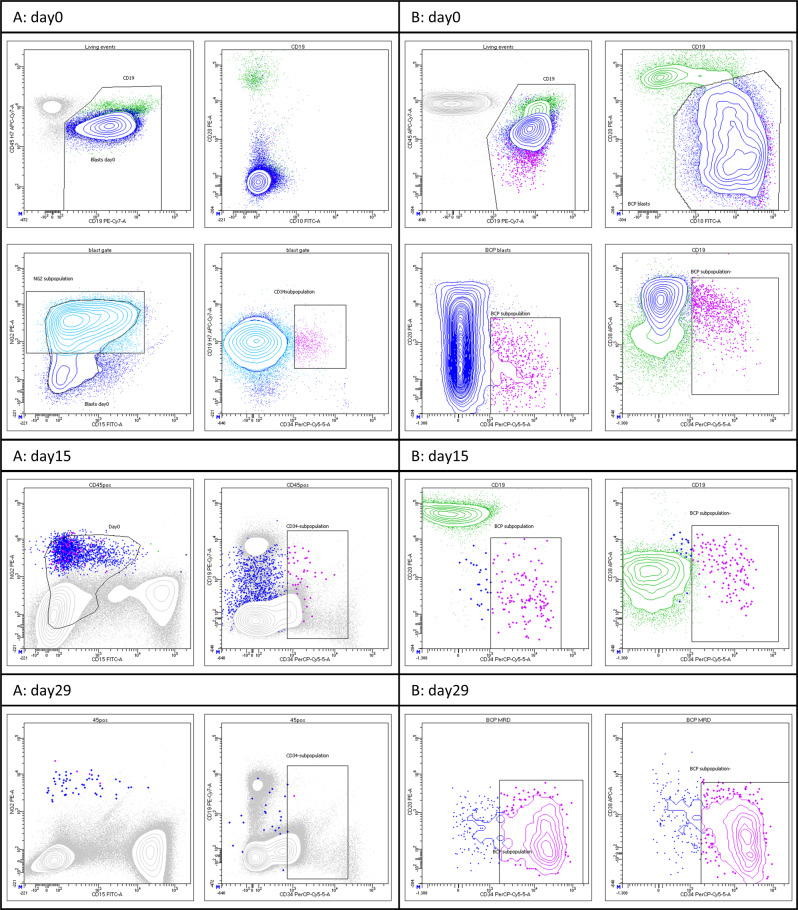
Two patient cases discrepantly stratified by FCM and PCR. **A** *KMT2A*-r BCP-ALL. Classically CD10neg/CD20neg with broad CD19 expression at diagnosis, where the majority of blasts exhibit NG2 expression and a smaller subpopulation CD34pos expression. During treatment there is partial loss of CD19 expression. For days 15 and 29, gating (blue) on blasts is done on NGSpos/CD33neg MRD population (plot not showed). Day 15 FCM-MRD = 1.08% and day 29 FCM-MRD = 0.01% versus day 29 PCR-MRD 1.0%, indicating NG2 only partly informative/additional NG2 loss during treatment in addition to CD19 decreased expression. **B** BCP-ALL exhibiting CD10pos and CD20broad expression at diagnosis with a smaller subpopulation, amounting to ~3% of the total blast population, exhibiting CD34 expression. During treatment there is a distinct treatment response on the CD34neg dominating subpopulation but continued persistence of the CD34pos subpopulation displaying a poor treatment response; day 15 FCM-MRD = 0.3% and day 29 FCM-MRD = 0.32% with decreasing CD38 expression and increasing CD34 expression. Neither of the two PCR markers used were able to detect the CD34pos subpopulation (day 29 PCR-MRD undetectable, QR 3 × 10^−5^), comprising too low a fraction of total blasts at diagnosis to be identified when screening for TCR/Ig gene rearrangements.

Discrepant results were obtained in 44 samples (Table S4). The FCM- and PCR-MRD led to discrepant EOI stratification in 33 patients (28 with FCM <10^−3^, five with PCR <10^−3^). Six of these experienced a relapse, all of whom had FCM-MRD <10^−3^. Four out of these six patients (1.2% of total) were stratified as SR patients based on the FCM-MRD result. In one of the six patients, no MRD was detected by FCM, which might be explained by the lack of a fully informative LAIP although the sensitivity was 2.9 × 10^−5^. In the other five patients with discrepant results and relapse, FCM-MRD was detected and ranged between 10^−3^ and 10^−4^. Marker modulation was observed in two of these patients, one of which had *KMT2A*-r with loss of CD19 expression (Fig. 6A). Insufficient hemolysis compromising the analysis was observed in 2, while in one patient no cause could be identified. Among the 44 discrepantly stratified cases, *KMT2A*-r (*n* = 5, *p* = 0.02) and B-other (*n* = 25, *p* = 0.001) were overrepresented.

PCR-MRD-related causes for discrepancy included poor sensitivity and/or QR and the presence of subclones that were not identified at diagnosis and became dominant during treatment (Fig. 6B).

## Discussion

The NOPHO ALL2008 study is the first to apply FCM-MRD-guided risk stratification of patients with BCP-ALL in a large, multi-MRD-center setting. We demonstrate excellent patient outcomes, comparable to protocols using PCR‐MRD-based stratification [4], with an EFS_5y_ of 86.1% and a CIR_5Y_ of 9.5% in a mixed cohort of children and adults. Furthermore, >96% of patients could be followed by FCM-MRD and an informative MRD marker was obtained for >99.8% of patients with relevant diagnostic material when combining FCM- and PCR-MRD.

The early (day 15) FCM-MRD response associated with relapse independent of the EOI response. This is in line with Basso et al., who studied 815 patients with FCM-MRD performed on day 15 of induction after a 7-day prednisolone monotherapy [29], but in contrast with Short et al., who examined the day 14 response on EFS in 389 adult ALL patients [30]. This discrepancy could be due to cutoff level variations, or to EFS versus CIR as outcome. We further found that a higher percentage of adults and children >10 years had a poor early response, in particular patients with B-other ALL. Accordingly, age-related differences in MRD levels have been shown also at EOI by us and others [23, 31].

We previously reported a poor EFS_5y_ for adult ALL-IR patients [23]. Here we show that high MRD on day 29 in B-other patients is more common in adults and is associated with a poor outcome. Notably, we found that all relapses among the adult ALL-IR patients occurred in the B-other subgroup. The higher relapse rate in this subgroup is likely due to the higher proportion of HR BCP-ALL disease entities such as Ph-like and *MEF2D*-rearranged BCP-ALL [32–34], which are more common in adults and have a poor outcome with conventional chemotherapy [35, 36]. Likewise, the association of age and high MRD on day 15 can be explained by the disease biology of B-other. Including testing for HR BCP-ALL genetics might improve prognosis for these patients by allowing the tailoring of MRD cutoff levels by genetics [37, 38] and by adding tyrosine kinase inhibitors to conventional therapy [39].

For SR/IR patients, the late (day 79) MRD response even at levels below 10^−3^ (above which patients were stratified to HSCT) was clearly associated with relapse, suggesting stratification with a lower cutoff could be of value. This is consistent with Borowitz et al. who found 10^−4^ to discriminate well with regard to EFS in 186 patients at week 12, albeit without MRD-based stratification at this timepoint, thus including patients with MRD ≥10^−3^ in the patient group with MRD ≥10^−4^ [21]. In HR patients, 10^−3^ [2], 5 × 10^−4^ [20], and 10^−4^ [3, 40] have previously been suggested as cutoff levels for late response. We had few patients with MRD of 10^−4^–10^−3^ after the second HR-block, but there was no difference in outcome for patients with MRD <10^−4^ and undetectable MRD, with a CIR_5Y_ of 17% for both.

The proportion of discrepant cases (FCM_det_/PCR_det_ and PCR_det_/FCM_det_) declined from the early to later timepoints, corresponding to more patients having a low/undetectable MRD by one or both methods as treatment progressed. We found a day 29 FCM_det_/PCR_det_ similar to what Theunissen et al. found for a threefold higher number of acquired cells [41]. The low day 15 FCM_det_/PCR_det_ (Fig. S1) could be explained by the relatively low number of cells acquired on day 15, Thus, the predictive effect of low/undetectable MRD by FCM is likely underestimated in this cohort, and further studies on an early identification of the very good responders are warranted.

The EOI FCM-MRD level was closely associated with relapse risk, regardless of other risk factors, and a stratification cutoff level of 10^−3^ was appropriate for the bulk of patients stratified to SR/IR. Further, only four patients stratified to SR by FCM-MRD at EOI, but to IR by PCR-MRD, experienced relapse. Notably, only three centers routinely performed both FCM- and PCR-MRD, and so the FCM-MRD prognostic value in comparison to PCR was likely underestimated, since PCR-MRD was only performed in the remaining centers, when the FCM-MRD was not informative. Our review of the discrepant cases revealed a not fully informative/heterogeneous LAIP, insufficient number of cells, and marker modulation (including CD19 loss in *KMT2A*-r cases) to be common reasons for an underestimation of the MRD by FCM. An association between cytogenetic subgroup and LAIP quality was also observed for the high hyperdiploid cases, who were underrepresented among patients with a non-informative LAIP. This is likely due to the frequent overexpression of CD123 in this group [42], as CD123 is a very informative marker for FCM-MRD monitoring [42].

PCR-MRD is a sensitive, standardized method with extensive, published data for risk stratification in BCP-ALL, but challenges remain. Not all patients have suitable PCR markers for monitoring, loss/gain of subclones are difficult to discover as PCR-MRD does not provide biological insight, QR and sensitivity are firmly limited by input DNA amount, and early (day 15) monitoring is not possible, as markers are not yet ready for implementation [6]. FCM-MRD is fast, provides information on intra-leukemic heterogeneity, and allows for monitoring of patients without gene rearrangement, thus solving most challenges of PCR-MRD. Disadvantages of FCM-MRD are the limited sensitivity, high analytical expertise, and lack of informative markers in some patients. However, these could be improved by the acquisition of more cells [41] and implementation of broader panels, and perhaps in time with technical developments like spectral FCM and bioinformatics. In this protocol, the median EOI FCM-MRD sensitivity was 4.7 × 10^−5^, corresponding to only 215,000 analyzed cells, and future protocols are likely to show an even better predictive value of FCM-MRD. It is therefore crucial to keep performing FCM-MRD and PCR-MRD in combination to continuously assess the value of both, as technological progress is made.

The combination of MRD methods in clinical protocols will also be a topic of discussion as HTS-MRD gains ground. HTS-MRD shows promise as a highly sensitive method for MRD-monitoring [11] and a potential replacement of PCR-MRD, as both methods detect TCR/Ig gene rearrangements. However, HTS-MRD is yet to be evaluated prospectively in clinical studies and is currently not useful for patients without detectable gene rearrangements [11, 43]. Whether the use of leukemia-specific targets will eventually allow for HTS-MRD monitoring in these patients as well, or if FCM-MRD will still be needed, is unclear. The present study shows that combining MRD methods and stratifying by the most informative method allows for MRD monitoring in all patients and ensures that any “blind spots” of one can be compensated for by the other. However, sufficient bone marrow material for analysis is an important prerequisite of patient stratification to the very low risk group, regardless of the MRD method applied. Thus, the balance between reaching adequate MRD sensitivity and achieving the advantages of a simultaneous analysis by two methods is difficult to find, as the bone marrow cells for analysis at MRD timepoints are limited. Future studies involving monitoring by multiple methods are warranted to clarify this issue.

In conclusion, we demonstrate proof-of-concept for FCM-MRD as a primary stratification method in BCP-ALL in a multicenter setting. Clinical outcomes are associated equally with FCM and PCR-MRD and the two methods in combination ensure an accurate monitoring of all patients with BCP-ALL. Future protocols using an increased number of acquired cells and 8–10 color panels will show whether FCM-MRD can reach the sensitivity necessary to also identify patients with a very low relapse risk. This would offer the potential to reduce therapy intensity in these patients. Also, more sensitive FCM-MRD could give additional prognostic information already on day 15, possibly allowing stratification at this timepoint.

## Supplementary information

Supplemental material

## References

[1] Zhou J, Goldwasser MA, Li A, Dahlberg SE, Neuberg D, Wang H (2007). Quantitative analysis of minimal residual disease predicts relapse in children with B-lineage acute lymphoblastic leukemia in DFCI ALL Consortium Protocol 95-01. Blood.

[2] Conter V, Bartram CR, Valsecchi MG, Schrauder A, Panzer-Grumayer R, Moricke A (2010). Molecular response to treatment redefines all prognostic factors in children and adolescents with B-cell precursor acute lymphoblastic leukemia: results in 3184 patients of the AIEOP-BFM ALL 2000 study. Blood.

[3] Gokbuget N, Kneba M, Raff T, Trautmann H, Bartram CR, Arnold R (2012). Adult patients with acute lymphoblastic leukemia and molecular failure display a poor prognosis and are candidates for stem cell transplantation and targeted therapies. Blood.

[4] Berry DA, Zhou S, Higley H, Mukundan L, Fu S, Reaman GH (2017). Association of minimal residual disease with clinical outcome in pediatric and adult acute lymphoblastic leukemia: a meta-analysis. JAMA Oncol.

[5] Borowitz MJ, Devidas M, Hunger SP, Bowman WP, Carroll AJ, Carroll WL (2008). Clinical significance of minimal residual disease in childhood acute lymphoblastic leukemia and its relationship to other prognostic factors: a Children’s Oncology Group study. Blood.

[6] Bruggemann M, Schrauder A, Raff T, Pfeifer H, Dworzak M, Ottmann OG (2010). Standardized MRD quantification in European ALL trials: proceedings of the Second International Symposium on MRD assessment in Kiel, Germany, 18-20 September 2008. Leukemia.

[7] Flohr T, Schrauder A, Cazzaniga G, Panzer-Grumayer R, van der Velden V, Fischer S (2008). Minimal residual disease-directed risk stratification using real-time quantitative PCR analysis of immunoglobulin and T-cell receptor gene rearrangements in the international multicenter trial AIEOP-BFM ALL 2000 for childhood acute lymphoblastic leukemia. Leukemia.

[8] Garand R, Beldjord K, Cave H, Fossat C, Arnoux I, Asnafi V (2013). Flow cytometry and IG/TCR quantitative PCR for minimal residual disease quantitation in acute lymphoblastic leukemia: a French multicenter prospective study on behalf of the FRALLE, EORTC and GRAALL. Leukemia.

[9] Pieters R, de Groot-Kruseman H, Van der Velden V, Fiocco M, van den Berg H, de Bont E (2016). Successful therapy reduction and intensification for childhood acute lymphoblastic leukemia based on minimal residual disease monitoring: Study ALL10 from the Dutch Childhood Oncology Group. J Clin Oncol.

[10] Vora A, Goulden N, Wade R, Mitchell C, Hancock J, Hough R (2013). Treatment reduction for children and young adults with low-risk acute lymphoblastic leukaemia defined by minimal residual disease (UKALL 2003): a randomised controlled trial. Lancet Oncol.

[11] Wood B, Wu D, Crossley B, Dai Y, Williamson D, Gawad C (2018). Measurable residual disease detection by high-throughput sequencing improves risk stratification for pediatric B-ALL. Blood.

[12] Gaipa G, Basso G, Biondi A, Campana D (2013). Detection of minimal residual disease in pediatric acute lymphoblastic leukemia. Cytom B Clin Cytom.

[13] Thorn I, Forestier E, Botling J, Thuresson B, Wasslavik C, Bjorklund E (2011). Minimal residual disease assessment in childhood acute lymphoblastic leukaemia: a Swedish multi-centre study comparing real-time polymerase chain reaction and multicolour flow cytometry. Br J Haematol.

[14] Neale GA, Coustan-Smith E, Stow P, Pan Q, Chen X, Pui CH (2004). Comparative analysis of flow cytometry and polymerase chain reaction for the detection of minimal residual disease in childhood acute lymphoblastic leukemia. Leukemia.

[15] Malec M, van der Velden VH, Bjorklund E, Wijkhuijs JM, Soderhall S, Mazur J (2004). Analysis of minimal residual disease in childhood acute lymphoblastic leukemia: comparison between RQ-PCR analysis of Ig/TcR gene rearrangements and multicolor flow cytometric immunophenotyping. Leukemia.

[16] Gaipa G, Cazzaniga G, Valsecchi MG, Panzer-Grumayer R, Buldini B, Silvestri D (2012). Time point-dependent concordance of flow cytometry and real-time quantitative polymerase chain reaction for minimal residual disease detection in childhood acute lymphoblastic leukemia. Haematologica.

[17] Denys B, van der Sluijs-Gelling AJ, Homburg C, van der Schoot CE, de Haas V, Philippe J (2013). Improved flow cytometric detection of minimal residual disease in childhood acute lymphoblastic leukemia. Leukemia.

[18] Fossat C, Roussel M, Arnoux I, Asnafi V, Brouzes C, Garnache-Ottou F (2015). Methodological aspects of minimal residual disease assessment by flow cytometry in acute lymphoblastic leukemia: a French multicenter study. Cytom B Clin Cytom.

[19] Stow P, Key L, Chen X, Pan Q, Neale GA, Coustan-Smith E (2010). Clinical significance of low levels of minimal residual disease at the end of remission induction therapy in childhood acute lymphoblastic leukemia. Blood.

[20] Ribera JM, Oriol A, Morgades M, Montesinos P, Sarra J, Gonzalez-Campos J (2014). Treatment of high-risk Philadelphia chromosome-negative acute lymphoblastic leukemia in adolescents and adults according to early cytologic response and minimal residual disease after consolidation assessed by flow cytometry: final results of the PETHEMA ALL-AR-03 trial. J Clin Oncol.

[21] Borowitz MJ, Wood BL, Devidas M, Loh ML, Raetz EA, Salzer WL (2015). Prognostic significance of minimal residual disease in high risk B-ALL: a report from Children’s Oncology Group study AALL0232. Blood.

[22] Maloney KW, Devidas M, Wang C, Mattano LA, Friedmann AM, Buckley P (2020). Outcome in children with standard-risk B-cell acute lymphoblastic leukemia: results of Children’s Oncology Group Trial AALL0331. J Clin Oncol.

[23] Toft N, Birgens H, Abrahamsson J, Griskevicius L, Hallbook H, Heyman M (2018). Results of NOPHO ALL2008 treatment for patients aged 1–45 years with acute lymphoblastic leukemia. Leukemia.

[24] Hrusak O, de Haas V, Stancikova J, Vakrmanova B, Janotova I, Mejstrikova E (2018). International cooperative study identifies treatment strategy in childhood ambiguous lineage leukemia. Blood.

[25] Modvig S, Madsen HO, Siitonen SM, Rosthoj S, Tierens A, Juvonen V (2019). Minimal residual disease quantification by flow cytometry provides reliable risk stratification in T-cell acute lymphoblastic leukemia. Leukemia.

[26] van der Velden VH, Panzer-Grumayer ER, Cazzaniga G, Flohr T, Sutton R, Schrauder A (2007). Optimization of PCR-based minimal residual disease diagnostics for childhood acute lymphoblastic leukemia in a multi-center setting. Leukemia.

[27] van der Velden VH, van Dongen JJ (2009). MRD detection in acute lymphoblastic leukemia patients using Ig/TCR gene rearrangements as targets for real-time quantitative PCR. Methods Mol Biol.

[28] Lin DY, Wei LJ, Ying Z (1993). Checking the Cox model with cumulative sums of martingale-based residuals. Biometrika.

[29] Basso G, Veltroni M, Valsecchi MG, Dworzak MN, Ratei R, Silvestri D (2009). Risk of relapse of childhood acute lymphoblastic leukemia is predicted by flow cytometric measurement of residual disease on day 15 bone marrow. J Clin Oncol.

[30] Short NJ, Kantarjian HM, Sasaki K, Cortes JE, Ravandi F, Thomas DA (2016). Prognostic significance of day 14 bone marrow evaluation in adults with Philadelphia chromosome-negative acute lymphoblastic leukemia. Cancer.

[31] Schafer ES, Hunger SP (2011). Optimal therapy for acute lymphoblastic leukemia in adolescents and young adults. Nat Rev Clin Oncol.

[32] Roberts KG, Li Y, Payne-Turner D, Harvey RC, Yang YL, Pei D (2014). Targetable kinase-activating lesions in Ph-like acute lymphoblastic leukemia. N Engl J Med.

[33] Den Boer ML, van Slegtenhorst M, De Menezes RX, Cheok MH, Buijs-Gladdines JG, Peters ST (2009). A subtype of childhood acute lymphoblastic leukaemia with poor treatment outcome: a genome-wide classification study. Lancet Oncol.

[34] Gu Z, Churchman M, Roberts K, Li Y, Liu Y, Harvey RC (2016). Genomic analyses identify recurrent MEF2D fusions in acute lymphoblastic leukaemia. Nat Commun.

[35] Jain N, Roberts KG, Jabbour E, Patel K, Eterovic AK, Chen K (2017). Ph-like acute lymphoblastic leukemia: a high-risk subtype in adults. Blood.

[36] Roberts KG, Gu Z, Payne-Turner D, McCastlain K, Harvey RC, Chen IM (2017). High frequency and poor outcome of Philadelphia chromosome-like acute lymphoblastic leukemia in adults. J Clin Oncol.

[37] O’Connor D, Enshaei A, Bartram J, Hancock J, Harrison CJ, Hough R (2018). Genotype-specific minimal residual disease interpretation improves stratification in pediatric acute lymphoblastic leukemia. J Clin Oncol.

[38] Enshaei A, O’Connor D, Bartram J, Hancock J, Harrison CJ, Hough R (2020). A validated novel continuous prognostic index to deliver stratified medicine in pediatric acute lymphoblastic leukemia. Blood.

[39] Harvey RC, Tasian SK (2020). Clinical diagnostics and treatment strategies for Philadelphia chromosome-like acute lymphoblastic leukemia. Blood Adv.

[40] Bassan R, Spinelli O, Oldani E, Intermesoli T, Tosi M, Peruta B (2009). Improved risk classification for risk-specific therapy based on the molecular study of minimal residual disease (MRD) in adult acute lymphoblastic leukemia (ALL). Blood.

[41] Theunissen P, Mejstrikova E, Sedek L, van der Sluijs-Gelling AJ, Gaipa G, Bartels M (2017). Standardized flow cytometry for highly sensitive MRD measurements in B-cell acute lymphoblastic leukemia. Blood.

[42] Djokic M, Bjorklund E, Blennow E, Mazur J, Soderhall S, Porwit A (2009). Overexpression of CD123 correlates with the hyperdiploid genotype in acute lymphoblastic leukemia. Haematologica.

[43] Wu D, Sherwood A, Fromm JR, Winter SS, Dunsmore KP, Loh ML (2012). High-throughput sequencing detects minimal residual disease in acute T lymphoblastic leukemia. Sci Transl Med.

