# Supplementary material for: Value of flow cytometry for MRD-based relapse prediction in B-cell precursor ALL in a multicenter setting
Source: Leukemia. 2020 Dec 14;35(7):1894–906. doi: 10.1038/s41375-020-01100-5 (PMC8257490; doi:10.1038/s41375-020-01100-5)
Supplement: Supplementary file 1 — Supplemental material [file 41375_2020_1100_MOESM1_ESM.pdf]

Supplementary material

Tube	FITC	PE	PerCPcy5.5	PE-Cy7	APC	APC-Cy7/ APCH7/ APC-Alexa Flour 750
**1**	CD10	CD20	CD34	CD19	CD38	CD45
**Clone**	HI10a or ALB1	L27 or B9E9	581 or 8G12	SJ25C1 or J3.119	HB-7	2D1
**2**	CD66c	CD123	CD34	CD19	CD22	CD45
**Clone**	KOR-SA3544	9F5	581 or 8G12	SJ25C1 or J3.119	SHCL-1	2D1
**3**	TdT	CD58	CD34	CD19	CD10	CD45
**Clone**	HT-6	1C3	581 or 8G12	SJ25C1 or J3.119	HI10a	2D1

Supplementary Table S1 NOPHO ALL2008 protocol obligatory combinations for BCP-ALL at diagnosis and follow-up. Antibody clones and conjugates in tubes 1-3 were mandatory, vendors were optional.

FCM/PCR	PCR available	No sample	No DNA	No clonal rearrangement	No QR<=10e^{-3}	PCR not analyzed	Other technical reason for no PCR	PCR Missing	All FCM
**Informative**	436 (42)	6 (0)	2 (0)	3 (0)	11 (2)	76 (6)	8 (1)	662 (40)	1204 (91)
**Non-informative**	26 (2)	0 (0)	0 (0)	2 (0)	0 (0)	0 (0)	0 (0)	3 (0)	31 (2)
**Partially informative**	102 (7)	0 (0)	1 (0)	2 (0)	1 (0)	7 (1)	1 (0)	81 (9)	195 (17)
**FCM not available**	5 (0)	3 (0)	0 (0)	1 (0)	0 (0)	0 (0)	1 (0)	0 (0)	10 (0)
**Other technical reason for no FCM**	0 (0)	0 (0)	0 (0)	0 (0)	0 (0)	0 (0)	0 (0)	3 (0)	3 (0)
**FCM missing**	12 (0)	0 (0)	0 (0)	0 (0)	0 (0)	0 (0)	0 (0)	32 (3)	44 (3)
**All PCR**	581 (51)	9 (0)	3 (0)	8 (0)	12 (2)	83 (7)	10 (1)	781 (52)	1487 (113)

Supplementary table S2 FCM- and PCR markers at diagnosis

FCM: Flow cytometry-based minimal residual disease. PCR: PCR-based minimal residual disease. QR: quantitative range. Relapse number in parenthesis.

Method/Timepoint	Day 15	Day 29	Day 79	After 2. HR block
**FCM sensitivity**	7.8x10^{-5}<br>(4.2 x10^{-5}-1.6 x10^{-4})	4.7 x10^{-5}<br>(3.0-7.9 x10^{-5})	3.8 x10^{-5}<br>(2.5-5.6 x10^{-5})	2.9 x10^{-5}<br>(2.4-4.5 x10^{-5})
**PCR QR**	1x10^{-4}<br>(1-3x10^{-4})	1x10^{-4}<br>(1-3x10^{-4})	1x10^{-4}<br>(1-5x10^{-4})	1x10^{-4}<br>(1-5x10^{-4})
**PCR sensitivity**	3 x10^{-5}<br>(1x10^{-5}- 1x10^{-4})	1 x10^{-5}<br>(1x10^{-5}- 1x10^{-4})	1 x10^{-5}<br>(1x10^{-5}- 1x10^{-4})	1 x10^{-5}<br>(1x10^{-5}- 1x10^{-4})

All values are given as median (IQR)

Supplementary Table S3 - FCM- and PCR-MRD sensitivity/QR at stratification timepoints

FCM: flow cytometry. HR: high risk. PCR: polymerase chain reaction. QR: quantitative range

	FCM undetectable/ PCR detectable	FCM detectable/ PCR undetectable	FCM<10^{-3}/ PCR≥10^{-3}	FCM≥10^{-3} /PCR<10^{-3}
**1.Marker modulation with loss of LAIP**	1		4	
**2.Not fully informative/heterogeneous LAIP at diagnosis**	1	2	7	
**3.Insufficient number of cells for FCM-MRD**			4	
**4.FCM technical limitations**	1	1	2	1
**5.Inclusion of false positive events**		3		
**6.PCR technical limitations**		2	1	1
**7.Cause unidentified. Specific and sensitive LAIP and PCR marker**	1	1	8	
**8.Discrepant around cut-off but difference less than 0.0001 to cut-off**			2	1

Supplementary Table S4 – Review of discrepantly stratified cases at end of induction by FCM and PCR

Causes for discrepant FCM and PCR results in 44 patients at end of induction. Discrepancy of FCM- and PCR-MRD at day 29 was defined as either a discordant detectable/undetectable or discrepant stratification by the day 29 cut-off level of 10^{-3}. The former was defined as an undetectable MRD by one method but detectable ≥10^{-4} by the other with a >2.5-fold higher value than the detection limit of the method with an undetectable MRD to account for individual variation in the detection limit of the two. Marker modulation with loss of LAIP included *KMT2A*-r with loss of CD19 lineage marker (illustrated in Figure 3A). FCM technical limitations included hemodilution, many regenerating granulocytes, suboptimal panel (incl. 4 colours), and insufficient red cell lysis in membrane tubes. PCR technical limitations included a sensitivity >10^{-3} and the presence of a CD34pos subpopulation at diagnosis, not detected by PCR, expanding at MRD timepoints leading to MRD underestimation (illustrated in Figure 3B).

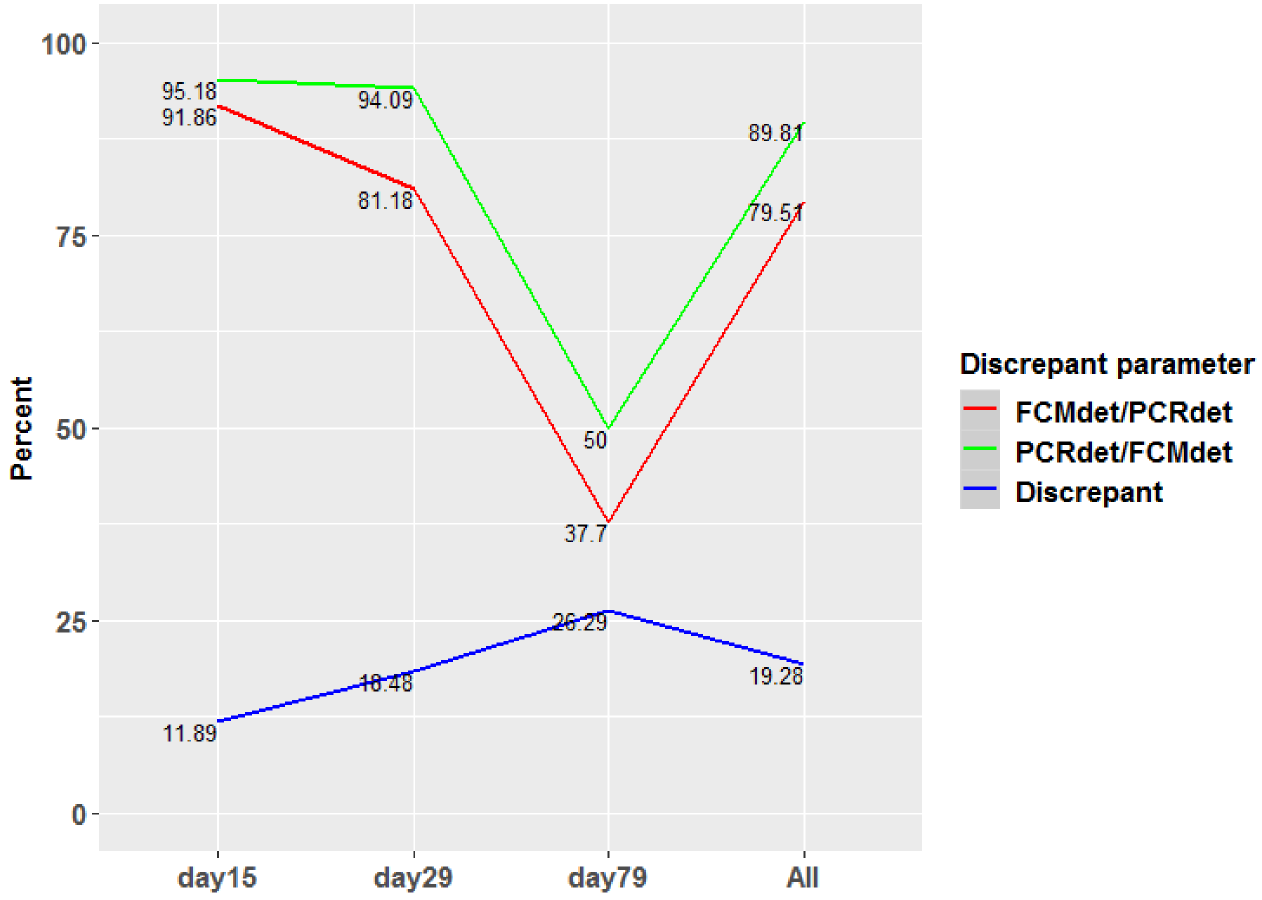

Supplementary figure S1 – Patients with discrepant detectable/undetectable MRD by FCM and PCR

The proportion given in percent of patients with a detectable FCM-MRD out of all patients with detectable PCR-MRD (FCMdet/PCRdet) and that of patients with detectable PCR-MRD out of all patients with detectable FCM-MRD (PCRdet/FCMdet) as well as that of patients with discrepantly detectable/undetectable (detectable by one method and undetectable by the other) samples at stratifying timepoints. FCM-MRD: flow cytometry-based minimal residual disease. PCR-MRD: polymerase chain reaction-based minimal residual disease.

BCP Supplementary Methods - FCM data analysis and gating strategy

FCM-based MRD detection of aberrant antigen expression was run in all NOPHO centers if an informative FCM marker combination was available at diagnosis. Initial diagnosis was performed according to WHO classification at local laboratories with any panel of choice. FCM-MRD evaluation in the context of the NOPHO ALL2008 study was only performed in NOPHO-approved MRD laboratories all being members of the Nordic Flow Cytometry group (NFCG). A QC program by NFCG included a yearly assessment of FCM files of BCP-ALL and T-ALL cases as previously described[13]. The results of this program were discussed at yearly meetings, also serving as a forum for the exchange of protocol updates and experience. All NFCG laboratories were required to participate in the QC rounds and the meetings. All NOPHO laboratories were also affiliated to the UK Neqas ALL program. The decision to use FCM-MRD as primary stratification method was based on data from the NOPHO ALL2000 protocol, using a 4-color standardized BCP-ALL FCM-MRD panel, showing slightly better event-free survival (EFS) prediction at a cut-off level of 10-3 compared to PCR (unpublished data). Further, all 18 NOPHO FCM-MRD centers in the Nordic/Baltic countries were in 2008 able to fulfil the protocol requirements developed by the NFCG in a standardized 6-color FCM-setting.

For identification of LAIPs for FCM-MRD evaluation, bone marrow (BM) samples were analyzed at diagnosis by the NOPHO MRD laboratories using the NOPHO ALL2008 protocol-defined standardized 6-colour MRD antibody combinations. The MRD antibody panel (Table S4) included immature B-lineage markers (CD10, CD34, TdT) and common B-lineage markers (CD19, CD20, surface CD22) in addition to CD38 and potentially aberrantly expressed MRD markers such as CD58, CD66c and CD123. In the MRD panel CD19, CD34 and CD45 were used as backbone markers.

For a complete classification of the leukemia, additional B-lineage (kappa, lambda, cytoplasmic CD22, cytoplasmic CD79α, cytoplasmic IgM, HLA-DR), T-lineage (cytCD3, CD2, CD7) and myeloid lineage (CD13, CD15, CD33, CD117, cytoplasmic MPO) antigens were analyzed with any panel of choice. Mixed phenotype acute leukemia (MPAL) and Acute undifferentiated leukemia (AUL) was excluded. At least 30,000 events were acquired at diagnosis although, if material was available, 100,000 events per marker-combination was recommended to ensure optimal identification of subpopulations. Normal cells present in BM samples served as positive and negative controls of antibody-reagent performance. The antigen expression of each marker was recorded as positive when >20% marker positive blasts were found and additionally recorded as dim, normal/medium, or bright, when fluorescence intensity (FI) was weaker, equal to or stronger compared to normal BM cells. In addition, immunophenotypic subpopulations were documented by recording bimodal or heterogenous marker expression. A leukemia-associated immunophenotype (LAIP) was categorized according to the following guidelines: If at least one marker was aberrantly expressed by >

90% of the leukemic BCP-lymphoblasts, the LAIP was considered as fully informative; if one or more markers were aberrantly expressed on part of the leukemic BCP-lymphoblasts or only on some but not all subpopulations of the leukemic BCP-lymphoblasts the LAIP were considered as partially informative. Cases in which no aberrant marker expression was detected at diagnosis were considered as non-informative.

BM samples taken at the MRD timepoints were analyzed using the standardized MRD antibody combinations. If cross-lineage antigen expression (e.g. myeloid or T-lineage marker expression) was detected at diagnosis, MRD monitoring was supplemented with a patient-tailored antibody combination. At MRD time points, at least 300.000 events, but preferably 1 million events, per antibody combination were analyzed when sufficient material was available.

The gating strategy according to NOPHO guidelines was as follows: firstly dead cells/debris were excluded based on the dot plots FCS/SSC and doublets were removed on the plots FSC Area/FSC Height as shown (Supplementary Figure S1); secondly, B-lineage CD19 positive events were selected on the CD45/CD19 and/or CD19/SSC dot plots depending on the expression level of CD45 and CD19; BCP-lymphoblasts could most often be distinguished from mature B-cells by negative/dim expression of CD20 and pos expression of CD10 at time of diagnosis as well as MRD timepoints. Subsequently, LAIP expression of BCP-lineage markers or cross-lineage markers was identified. Antigen marker modulation on the BCP-lymphoblasts may occur during treatment and should be taken into account during data analysis. For further verification of the gated BCP-ALL blasts, the LAIP clustering events were back-gated into dot plots with other antibody combinations including CD45/CD19 and FSC/SSC plots, keeping in mind that CD45 expression often increases during the course of therapy. To report detectable MRD, a cluster of cells (at least 10 events) with aberrant immunophenotype and adequate FSC/SSC properties had to be identified. However, the results were considered more quantifiable if 30-40 LAIP events were detected, and more credible when comparable results were identified in more than one antibody combination. If the data were equivocal, the reason for doubts should be reported. The final MRD value was considered as the highest of the values obtained from the results of the most informative antibody combinations, clearly resolving abnormal blasts from normal BM cells. All data were reported into the NOPHO ALL2008 MRD database.
